# Notes and News

**Published:** 1904-12-31

**Authors:** 


### Notes and News.

The Société de Chirurgie of Paris has appointed a special committee to study the results of Dr. Doyen's method of treating cancer. The members are Drs. Berger Nelaton Kermesson and Monod and a medical reporter.

The abandoned observatory on the summit of Ben Nevis might be taken up by some enterprising person as a health resort. The staff of the observatory is reported to have enjoyed excellent health, and it was only when they returned to sea-level that they contracted colds.

Dr. E. Stokes, who has been appointed medical officer and bacteriologist to the Sydney Metropolitan Board of Water Supply and Sewage, obtained his diploma of Public Health at the Royal Colleges of Physicians and Surgeons, passing with honours. He is a graduate of the University of Sydney.

It is reported that the street toy-sellers of Ludgate Hill made a special business for a week before Christmas on behalf of the Crippled Children's Christmas Dinner Fund, and they also each contributed a toy for the children. The true spirit of charity, entailing willing personal sacrifice, prompted these gifts of the poor to the poor.

One of the Medical Societies of Paris has taken in hand the question of the sale of food from street stalls. The Society maintains that the food thus exposed is open to serious dangers. It has passed several resolutions to insure all possible precautions being taken, and has gone as far as to recommend that the food exhibited should undergo periodical examination in the municipal laboratory. It would appear that the existence of street stalls is threatened in France as well as in England. It means a sacrifice of convenience to hygiene.

By the King's consent the Edinburgh Victoria Hospital for Consumption may use the prefix of "Royal" in future.

The late Mr. Herbert Allingham, F.R.C.S., has bequeathed £2,000 to St. George's Hospital to found a Herbert Allingham Surgical Scholarship.

At a large meeting at Guildford in aid of the Royal Surrey County Hospital, a donation of £500 from the High Sheriff, Sir E. Stern, was announced.

The Federal Chamber of Manufacturers of Victoria are endeavouring to prevent the importation of proprietary medicines which do not define such poisons as enter into their composition.

The American National Association for the Prevention of Tuberculosis, which holds its first anniversary in Maquest, is to appoint an advisory committee of medical practitioners and laymen, and to make a public appeal throughout the United States.

Dublin is to have a new hospital, through the will of the late Mrs. Lewis, of Kilcullen, who has bequeathed £80,000 for the purpose. This will add one more to the already more than sufficient number of small hospitals in Dublin. The institution is to be a memorial, and will be called the Hervey Lewis Memorial Hospital.

We sincerely hope that the offer made by Mr. Donkin of Wimbledon to contribute £250 to the North-Eastern Hospital for Children will be possible of fulfilment. The condition attending the offer is that five other donations of similar amount must be forthcoming by the end of the year. Two generous friends have already come forward, and although the time is short and the calls upon resources are many, we trust to announce success.

A letter in the *Times* of Monday last gives an interesting account of the arrangements made by the Japanese Red Cross Society in the matter of hospital ships. The society has arranged to build its own ships to its own requirements, and to sell them to a steamship company with the proviso that they shall be at the service of the society when required, for a stated rental whilst in use. There are many other clauses in the scheme, which appears to be a practical one, and to have worked well. The writer of the letter quoted from concludes by saying:—"Could not Great Britain take a lesson from Japan in this respect? While it may not be possible for the British Red Cross Society, which does not compare with the Japanese society, to undertake the work, the War Office might well enter into an arrangement with one of the great British steamship lines somewhat upon the model of the Japanese arrangement. The cost would be very small, and the advantages so great that the idea is at least worth consideration"

